# Olive Oil Sensory Analysis as a Tool to Preserve and Valorize the Heritage of Centenarian Olive Trees

**DOI:** 10.3390/plants11030257

**Published:** 2022-01-19

**Authors:** Nuno Rodrigues, António M. Peres, Paula Baptista, José Alberto Pereira

**Affiliations:** Centro de Investigação de Montanha (CIMO), Campus de Santa Apolónia, Instituto Politécnico de Bragança, 5300-253 Bragança, Portugal; peres@ipb.pt (A.M.P.); pbaptista@ipb.pt (P.B.); jpereira@ipb.pt (J.A.P.)

**Keywords:** Côa Valley, descriptive sensory profile, statistical tools, differentiation

## Abstract

In inland areas of Portugal and some regions of the Mediterranean basin, olive production is based on traditional olive groves, with low intensification, local cultivars, aged plants, and centenarian trees. These plants play a key role in the ecosystem, contributing to carbon sequestration and possessing a high genetic diversity, particularly important for selecting cultivars more resistant to climatic changes. Appreciation of the value of this genetic diversity implies genetic, morphological, and physicochemical characterization of centenarian trees, which is expensive and time-consuming. Sensory evaluation is also of utmost importance. Thus, in this study, centenarian olive trees were selected in the Côa Valley region, a UNESCO World Heritage site. The descriptive sensory profile of their extracted olive oils was established and used to cluster the oils, using hierarchical clustering analysis, and consequently the olive trees, into five groups with similar intensities of perceived olfactory–gustatory attributes. Each cluster revealed olive oils with unique sensory patterns, presumably due to similarities of the olive trees, confirming the potential of the proposed screening approach. The identification of sensorially homogeneous oil-tree groups would reduce the number of specimens needed for subsequent morphological, genetic, and chemical characterization, allowing a cost-effective and robust future evaluation procedure.

## 1. Introduction

The olive tree is one of the most ancient cultivated crops in the Mediterranean basin. This plant is well-adapted to this region, where around 90% of the world’s production of olive oil is concentrated [1]. Apart from the economic and social importance of the olive sector, in the Mediterranean region, olive groves provide important ecosystem benefits. This is especially true of traditional orchards, with well-adapted local olive cultivars and aged plants, some of them centenarian [2,3]. The benefits generated by traditional olive groves need to be evaluated in a holistic framework related to the production of raw materials (olives, leaves, and wood) as well as other social, geographical, and environmental aspects [4]. In today’s climate change conditions, the contribution to carbon sequestration could be considered one of the most important of the ecosystem benefits provided by traditional olive groves. Due to their long life cycle, permanent fruit trees, such as centenarian olive trees, potentially sequester a high amount of atmospheric carbon accumulated in their organs, namely the trunk, branches, and roots [5]. Another ecosystem benefit provided by centenarian olive trees is their high genetic diversity, which is also particularly important for possible selection and adaptation to changes in climate [6,7]. The genetic diversity of the olive tree is vast [8,9,10], with a large number of analyzed cultivars, although only a few have a broad and worldwide distribution. However, in some areas of the Mediterranean region, the olive germplasm is under-studied. The northeast of Portugal, the second-most important region in the country with 82,767 hectares of olive groves and a production of 117,343 tons of olives in 2018 [11], is known for the high quality of its olive products. The main olive cultivars are Cobrançosa, Madural, Verdeal Transmontana, Cordovil, Santulhana, and Negrinha de Freixo [2,12], although several other minor and less-distributed cultivars exist. Nevertheless, many centenarian olive tree specimens in the region belong to unknown or unanalyzed cultivars, and suffer a high risk of disappearing. For this reason, olive tree germplasm characterization is urgent. This biodiversity analysis can take into account several factors. A survey of the morphological characteristics of olive organs (olive leaves, flowers, fruits, and endocarps) is usually carried out in complement to some aspects of plant behavior, from which genetic markers are usually used for the identification and characterization of olive cultivars [13,14,15]. In other cases, the characterization focuses on different parameters of interest, such as the yield, resistance to pests and diseases or drought conditions, adaptation to mechanical harvest, or chemical quality of the olive oil [16,17,18,19]. When it is intended to value centenarian olive tree specimens, the search for differentiated olive oils with specific and desired chemical and sensory attributes is usually taken into account. For example, the search for specimens with high amounts of antioxidants, such as phenolic compounds and tocopherols [20,21], as well as exceptional sensory properties, have been explored [2]. Recently, some studies reported that genetic effects are the main source of variation for most olive oil constituents, leading to great variability in the composition of olive oils [22,23,24]. This aspect is correlated with the sensory profile of olive oils, assessment of which is mandatory according to European Union regulations for accurately establishing oil quality [25,26]. Thus, considering the relationship between the genetic component and sensory characteristics, the use of the sensory profile of olive oils together with statistical techniques can be seen as a practical and useful tool for identifying groups of plants (i.e., centenarian trees) with similar characteristics, reducing the number of unknown specimens that must be fully characterized. In this context, in this study, centenarian olive trees from the Côa Valley region (Northeast Portugal) were selected, and the sensory profiles of the extracted olive oils were evaluated by a sensory panel and further statistically analyzed to establish groups of olive oils with unique sensory characteristics, which in the future will allow identification of a reduced number of centenarian olive trees for systematic genetic, morphological, and physicochemical characterization.

## 2. Results and Discussion

From the 150 centenarian olive trees selected in the Côa Valley region (coded t1 to t150), olive oils were only extracted from the olives collected from each of 96 trees, from which a sufficient amount of olives could be harvested. Each oil was then analyzed, having verified that all of them fulfilled the legal thresholds [25] for extra virgin olive oil classification (free acidity lower than 0.8%, peroxide value lower than 20 mEq O_2_/kg, extinction coefficients at 232 and 268 nm lower than 0.22 and 2.50, respectively; data not shown). All oils were also evaluated by a sensory panel, establishing a descriptive sensory profile for each one. As shown in Table 1, the panelists perceived 32 positive sensations (13 olfactory attributes and 19 gustatory attributes), although several of them were only detected in a minority number (less than 50%) of the oils evaluated (e.g., olfactory: banana, cherry, plum, rosemary, lavender, and tomato leaves; gustatory: banana, kiwi, cherry, apricot, strawberry, plum, olive leaves, rosemary, and lavender). It should be noted that the perceived sensations, as well as the intensity ranges found, are, in general, in agreement with those reported in the literature for Moroccan and Tunisian olive oils [27,28], as well as for Portuguese oils extracted from minor cultivars of centenarian olive trees [2,20]. It has been reported that olive cultivar and genetic factors influence the sensory profile of extracted olive oils [28,29,30].

**Figure 1:**
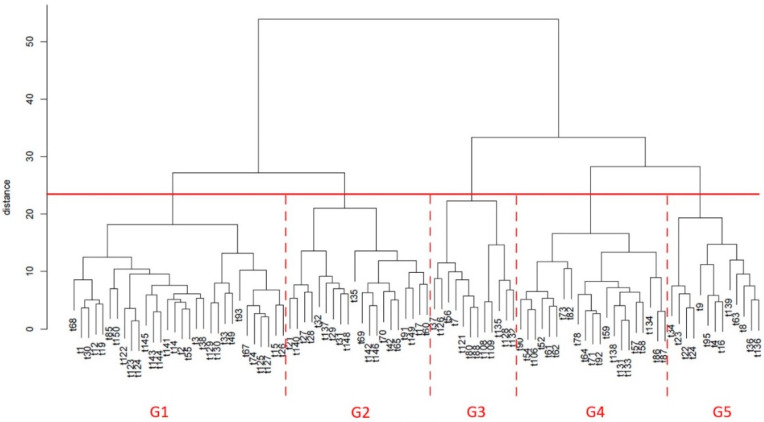
Dendrogram with the identification of five clusters/groups (G1, G2, G3, G4 and G5), for a Euclidean distance from 0 to 25, based on the dissimilarities of the sensory profiles of oils obtained from centenarian olive trees grown in the Côa Valley region.

As can be inferred from Table 1, the average CVr% for each perceived olfactory or gustatory attribute varied between 3.0% and 8.3%, with maximum values lower than 20%, which is the International Olive Council (IOC) threshold, confirming the evaluation skills of the trained panelists. The variability found in the sensory profiles of the 96 olive oils extracted from olives harvested from centenarian trees, as well as the wide range of intensities perceived by the panelists for each detected sensation, allowed the expectation that the oils, and thus the respective olive trees, could be clustered into different groups with a similar sensory pattern. The dendrogram obtained by hierarchical clustering analysis confirmed the possibility of splitting the 96 olive oils into different clusters/groups based on the dissimilarities found in the multi-dimensional sensory data established by the panelists (Figure 1). The dendrogram obtained using the sensory profiles (Figure 1) split the olive oils and, thus, the respective centenarian olive trees, into five main clusters (G1 to G5), with a Euclidean distance ranging from 0 to 25. Cluster G1 contained 30 olive oils/olive trees, G2 contained 20 olive oils, G3 contained 12 olive oils, G4 contained 21 olive oils, and G5 contained the other 13 olive oils. It should be noticed that all five clusters consisted of several subclusters, pointing out the variability in the sensory profiles of the studied olive oils.

**Figure 2:**
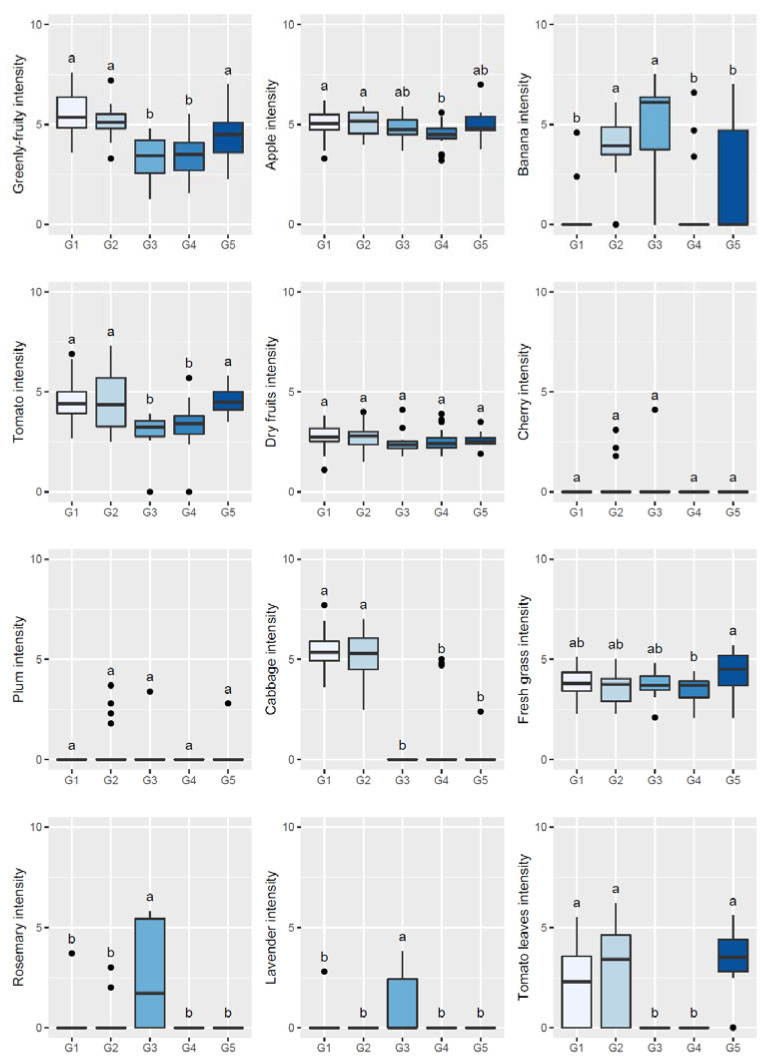
Sensory profile boxplots (olfactory sensations) found in olive oils extracted from olives from centenarian olive trees in the Côa Valley region. Different lowercase letters signify statistically significant differences at a significance level of 5% (one-way ANOVA followed by Tukey’s multi-comparison test).

**Figure 3:**
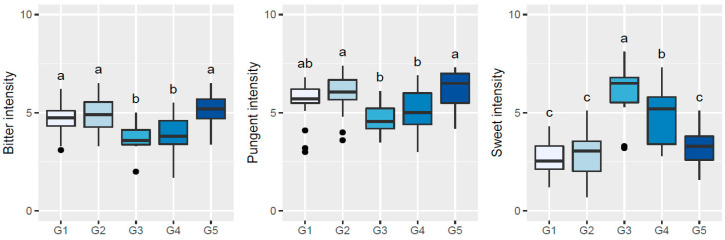
Sensory profile boxplots (bitter, pungent, and sweet intensity) found in olive oils extracted from olives from centenarian olive trees in the Côa Valley region. Different lowercase letters signify statistically significant differences at a significance level of 5% (one-way ANOVA followed by Tukey’s multi-comparison test).

**Figure 4:**
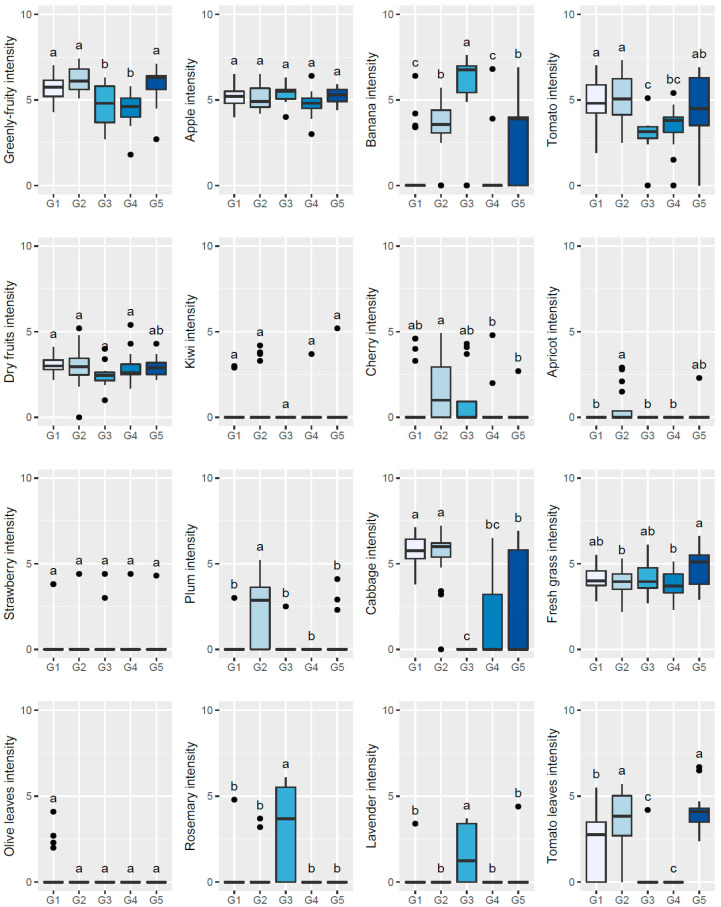
Sensory profile boxplots (gustatory sensations) found in olive oils extracted from olives from centenarian olive trees in the Côa Valley region. Different lowercase letters signify statistically significant differences at a 5% significance level (one-way ANOVA followed by Tukey’s multiple comparison test).

To further understand the sensory patterns of each of the abovementioned five clusters/groups of olive oils/olive trees, boxplots and one-way ANOVA were used to compare the olfactory (Figure 2) or gustatory (Figure 3 and Figure 4) sensations perceived among the five established groups of oils by the sensory panel. From those figures, it can be inferred that oils clustered in G1 showed high olfactory and gustatory intensities of greenly fruity sensations, with intense notes of tomato and cabbage olfactory–gustatory sensations in addition to high olfactory intensities of tomato leaves, possessing high bitter and pungent sensations and low sweetness. Oils belonging to cluster G2 were distinguished from the previous ones mainly due to the higher olfactory–gustatory intensity of banana, and the perceived gustatory fruit notes of cherry, apricot, plum, and tomato leaves attributes rather similar to the trends of the oils from G1. In contrast, oils from cluster G3 showed a lower olfactory–gustatory intensity of greenly fruity sensations, with lower bitterness and pungency, and a markedly higher sweetness; the perceived rosemary and lavender olfactory–gustatory intensities were probably responsible for the unique sensory pattern of these oils. Olive oils grouped within cluster G4 also possessed lower olfactory–gustatory intensities of greenly fruity as well as tomato sensations, showing a lower bitterness and pungency with high sweetness; the near absence of fruit and herbaceous notes was most likely responsible for their unique sensory fingerprint. Finally, oils from cluster G5, which from an overall sensory pattern were quite similar to those from clusters G1 and G2, can be distinguished from the other oils due to the slightly higher intensity of olfactory–gustatory sensations of fresh grass and lower olfactory–gustatory intensities of cabbage sensations. Interestingly, fresh grass sensations have been described as a characteristic of olive oils extracted from olives from olive tree cultivars grown in northeast Portugal [2]. These findings clearly pointed out that the sensory profiles of olive oils from centenarian olive trees, together with hierarchical clustering analysis, may be used as a practical fingerprint approach to identify centenarian trees that would produce oils with unique sensory patterns, contributing to an appreciation of their value and protection of their exceptional and intrinsic genetic diversity.

## 3. Materials and Methods

### 3.1. Sampling

#### 3.1.1. Tree Selection and Harvest

**Figure 5:**
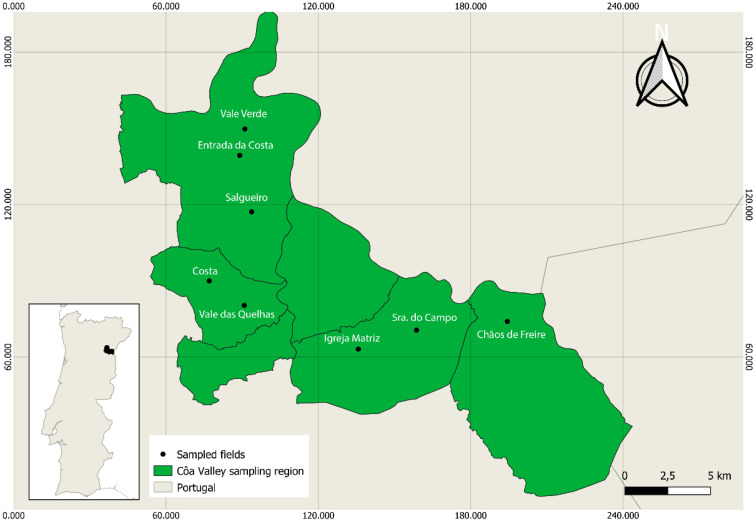
Côa Valley sampling region with the indication of sampling fields. Map projected in ETRS89/PT-TM06.

The experimental part of this work took place in 2020, in the Côa Valley region of northeast Portugal. Firstly, 150 centenarian olive tree specimens were selected, in eight distinct locations within the region: 14 in Chãos de Freire (Barca D’Alva), 18 in Senhora do Campo (Almendra), 10 in the Igreja Matriz (Almendra), 12 at Vale das Quelhas (Muxagata), 20 at Costa (Muxagata), 20 at Salgueiro (Vila Nova de Foz Côa), 20 at Entrada da Costa (Pocinho), and 30 in Vale Verde (Pocinho) (Figure 5). At each sampling point, the selection of the trees was based on their appearance, structure, trunk thickness, and information given by the local producers. In the harvest period, from the 150 initially selected trees, 96 were chosen because they produced at least 4–5 kg of fruits, allowing the extraction of a representative olive oil sample. Olives were harvested between two (MI 2) and three (MI 3) in the maturation index, which was determined according to IOC guidelines [31]. The fruits of each plant were extracted independently, making it possible to establish a direct relationship between oil and olive tree.

#### 3.1.2. Oil Extraction

The extraction of oils from each plant was performed as described by Rodrigues et al. [2]. The fruits were processed in the first 24 h after harvest, in a pilot extraction plant with an Abencor analyzer (Comercial Abengoa S.A., Seville, Spain) with three main units: a mill (hammer mill MM100 from MC2., Seville, Spain; with a 5.5 mm diameter screen and a 1.5 kW single-phase motor), a thermobeater (Thermo-Mixer TB-100 from MC2., Seville, Spain; with 8 working posts and 8 mixing jars with temperature regulation and individual propellers for mixing the paste), where malaxation takes place at controlled temperature, and a centrifuge (Centrifugal Machine CF-100 from MC2., Seville, Spain; with 1.5 kW three-phase motor, a stainless drum that rotates at 3500 rpm, and an automatic timer). Olives were milled, the paste was homogenized, and about 700 g was transferred to the thermobeater unit (20 min) for malaxation, using a thermostatic water bath at 25 °C. Then, the mixture was centrifuged and decanted, and the olive oil collected. For each sample (i.e., olive tree) at least four cups were prepared, and after extraction, the obtained olive oils from the same tree were mixed in the same bottle. Once the extraction process was finished, the oils were prepared for analysis, and filtered (Whatman paper no. 4) over anhydrous sodium sulfate to remove the solid particles and residual water. The olive oils were stored in 100 mL dark bottles and protected from light exposure at room temperature (20–25 °C).

### 3.2. Evaluation of Quality Parameters

Olive oils were analyzed according to European Union standard methods [25]. Following the above-mentioned EU regulation, a sensory panel with eight trained members (five men and three women, aged from 25 to 52 years old, with an average age of 37 years) evaluated all olive oil samples. The panel, from the Agriculture School of the Polytechnic Institute of Bragança, is a well-trained panel with more than five years of experience in sensory analysis of olive oil and table olives, and very familiar with olive oil sensory lexicon and assessment scales. All the analysis took place in a tasting room with standardized glasses, and all procedures followed International Olive Council (IOC) guidelines [32,33]. At the beginning of each session, an independent sample was taken to verify reproducibility. The descriptive profile was assessed using a test sheet with some modification, described by Rodrigues et al. [2], following the recommendations of the International Olive Council [34]. The olfactory intensities were graded using a continuous scale ranging from 0 (no perceived sensation) to 10 (maximum intensity of perceived sensation), and were used to assess the intensity and balance of fruity (mature or green) sensations, fruit sensations, and herbaceous sensations. The intensities of the gustatory–retronasal attributes were graded using a similar scale, evaluating the intensity and balance of fruity (mature or green), sweet, bitter, pungent, fruity, and herbaceous sensations. To establish the sensory profile and not influence the panelists, blank lines for identifying possible sensory descriptors were included in the test sheet. Any reference could be given to the expected attributes, and the trained panelists were free to select the attributes (descriptors) that they perceived during the sample’s sensory evaluation. Finally, the overall sensory perceptions were graded using a similar continuous scale, determining the complexity and the persistence of sensations. For complexity, the panel evaluated the combination of the different positive sensations perceived for each olive oil. A higher number of perceived sensations resulted in greater complexity. In contrast, a low number of sensations decreased the score of this parameter. In evaluating persistence, the panel ranked the perception of the different sensations that persist in the mouth over time. Long periods would mean a high persistence, and if the sensations disappeared, a low persistence was scored. The reproducibility of the panelists’ scores was evaluated based on the robust coefficient of variation (CVr%), calculated following the guidelines of the IOC (COI/T.20/Doc. No 15/Rev. 10 2018) [32].

### 3.3. Statistical Analysis

A hierarchical clustering dendrogram was applied to identify possible groups of olive oils/olive trees with similar sensory profiles (similar olfactory and gustatory attributes as well as perceived intensities). The hierarchical cluster analysis uses a set of dissimilarities for clustering n objects (olive oils/olive trees). Initially, each object is assigned to its own cluster, and then the algorithm proceeds iteratively, at each stage joining the two most similar clusters, continuing until there is just a single cluster. At each stage, distances between clusters were recomputed by the Lance–Williams dissimilarity formula, according to the Ward’s minimum variance method. The ward D2 algorithm was used, and so the dissimilarities between clusters were squared before cluster updating, computed for a limited number of distance/linkage combinations based on the squared Euclidean distance and centroid linkage. Boxplots of the sensory attributes were also used to visualize the variable’s dispersion within each group previously identified by the hierarchical clustering analysis. The 1st, 2nd (median), and 3rd quartiles were plotted together with the box bars that corresponded to the values comprised between the 1st and 3rd quartiles. Additionally, whiskers were plotted (1.5 × the inner quartile spread in length, measured from the median), establishing arbitrary cutoff points that allowed possible outside values to be identified. Minimum and maximum values that fell outside the whisker range were also plotted (dot symbols) and corresponded to possible extreme values or outliers. Finally, one-way ANOVA was also applied to verify the existence of statistically significant differences of each perceived sensory sensation among the established groups of olive oils/olive trees. When a statistically significant group effect was found, the Tukey’s post-hoc multiple comparison test was further applied to identify which groups were similar and which were different, at a 5% significance level. The analysis output was included within each boxplot, using lowercase letters; the same letter was used when no statistically significant difference was found, and different letters in the opposite case. The statistical analysis was performed using the free open-source statistical program R (version 3.6.2).

## 4. Conclusions

In the present work, olive oils obtained from 96 centenarian specimens from the Côa Valley region were characterized in terms of their sensory profile. The panelists perceived different positive sensory attributes, including rare fruit attributes such as plum, cherry, kiwi, apricot, and strawberry. Hierarchical clustering analysis of the olive oils’ sensory data identified five main clusters/groups, composed of several sub-clusters, comprising between 12 and 30 oils, corresponding to the same number of centenarian olive trees. The preliminary clustering verified that each main cluster had an unique overall sensory fingerprint, although it also showed the need to be complemented by other key information, such as morphological, genetic, and/or physicochemical data. Even so, the potential of the proposed sensory-statistical approach was verified, allowing a preliminary selection of centenarian olive trees that facilitate obtaining olive oils with specific and differentiated olfactory and gustatory sensations. Thus, the information gathered in this study contributes valuable knowledge regarding centenarian olive trees of the Côa Valley region, providing a basis for safeguarding these olive trees’ genetic heritage and their relevance. Moreover, it may be used to support the future selection of olive groves for new plantations, due to their sensory potential in delivering differentiated oils to the competitive olive oil market.

## Figures and Tables

**Table 1:** Olfactory and gustatory sensations perceived by the sensory panel in the 96 olive oils evaluated: sensation perceived, percentage of oils for which the sensation was perceived, minimum–maximum average intensity perceived, and related average (minimum–maximum) robust coefficient of variation (CVr%).

Sensation	Percentage of Oils with Perceived Sensation	Minimum–Maximum Average Intensities	Average (Minimum–Maximum) CVr%
*Olfactory sensations*			
Greenly fruity	100%	1.3–7.6	4.7 (0.0–17.2)
Apple	100%	3.2–7.0	3.3 (0.0–17.1)
Banana	38%	2.4–7.5	3.5 (0.0–12.2)
Tomato	98%	2.4–7.3	3.8 (0.0–15.2)
Dry fruits	100%	1.1–4.1	4.7 (0.0–17.6)
Cherry	4%	1.8–4.1	4.9 (1.9–10.7)
Plum	6%	1.8–3.7	7.0 (3.9–14.5)
Cabbage	56%	2.4–7.7	4.0 (0.0–14.2)
Fresh grass	100%	2.1–5.7	4.1 (0.0–15.1)
Rosemary	9%	2.0–5.8	3.4 (0.6–6.0)
Lavender	6%	2.1–3.8	7.0 (3.9–14.0)
Tomato leaves	44%	2.2–6.2	4.9 (0.0–15.7)
*Gustatory sensations*			
Sweet	100%	0.7–8.1	4.3 (0.0–14.9)
Bitter	100%	1.7–6.5	3.8 (0.0–17.9)
Pungent	100%	3.0–7.4	3.0 (0.0–11.1)
Greenly fruity	100%	1.8–7.4	3.7 (0.0–15.4)
Apple	100%	3.0–6.5	3.0 (0.0–11.6)
Banana	46%	2.5–7.6	4.1 (0.0–14.0)
Tomato	97%	1.5–7.3	3.8 (0.0–18.0)
Dry fruit	99%	1.0–5.4	4.8 (0.0–14.1)
Kiwi	8%	2.9–5.2	6.2 (0.9–18.1)
Cherry	21%	2.0–4.9	5.5 (0.0–18.5)
Apricot	6%	1.5–2.9	8.3 (2.3–17.5)
Strawberry	7%	3.0–4.4	3.3 (0.0–9.1)
Plum	17%	2.3–5.2	5.1 (2.2–12.3)
Cabbage	64%	2.6–7.2	3.2 (0.0–14.5)
Fresh grass	100%	2.2–6.6	3.9 (0.0–19.4)
Olive leaves	4%	2.0–4.1	5.5 (1.6–14.5)
Rosemary	10%	2.1–6.1	5.0 (0.6–11.4)
Lavender	8%	2.5–4.4	7.1 (1.0–10.5)
Tomato leaves	51%	1.8–6.7	4.9 (0.0–14.0)

## Data Availability

The data presented in this study are available on request from the corresponding author.
